# Nonclassic congenital adrenal hyperplasia due to 21-hydroxylase deficiency in women: diagnosis and treatment: Number 11 – 2024

**DOI:** 10.61622/rbgo/2024FPS11

**Published:** 2024-11-25

**Authors:** Andrea Prestes Nácul, Ana Carolina Japur Sá Rosa e Silva, Daniela Angerame Yela, Sebastião Freitas de Medeiros, José Maria Soares, Gabriela Pravatta Rezende Antoniassi, Lia Cruz da Costa Damásio, Técia Maria de Oliveira Maranhão, Gustavo Arantes Rosa Maciel, Cristina Laguna Benetti-Pinto

**Affiliations:** Grupo Hospitalar Conceição Hospital Fêmina Porto Alegre RS Brazil Hospital Fêmina, Grupo Hospitalar Conceição, Porto Alegre, RS, Brazil; Universidade de São Paulo Faculdade de Medicina de Ribeirão Preto Departamento de Ginecologia e Obstetrícia Ribeirão Preto SP Brazil Departamento de Ginecologia e Obstetrícia, Faculdade de Medicina de Ribeirão Preto, Universidade de São Paulo, Ribeirão Preto, SP, Brazil; Universidade Estadual de Campinas Faculdade de Ciências Médicas Departamento de Tocoginecologia Campinas SP Brazil Departamento de Tocoginecologia, Faculdade de Ciências Médicas, Universidade Estadual de Campinas, Campinas, SP, Brazil; Universidade Federal de Mato Grosso Departamento de Obstetrícia e Ginecologia Cuiabá MT Brazil Departamento de Obstetrícia e Ginecologia, Universidade Federal de Mato Grosso, Cuiabá, MT, Brazil; Universidade de São Paulo Faculdade de Medicina Hospital das Clínicas São Paulo SP Brazil Departamento de Obstetrícia e Ginecologia, Hospital das Clínicas, Faculdade de Medicina, Universidade de São Paulo, São Paulo, SP, Brazil; Universidade Estadual de Campinas Faculdade de Ciências Médicas Departamento de Tocoginecologia Campinas SP Brazil Departamento de Tocoginecologia, Faculdade de Ciências Médicas, Universidade Estadual de Campinas, Campinas, SP, Brazil; Universidade Federal do Piauí Departamento de Ginecologia e Obstetrícia Teresina PI Brazil Departamento de Ginecologia e Obstetrícia, Universidade Federal do Piauí, Teresina, PI, Brazil; Universidade Federal do Rio Grande do Norte Departamento de Tocoginecologia Natal RN Brazil Departamento de Tocoginecologia, Universidade Federal do Rio Grande do Norte, Natal, RN, Brazil.; Universidade de São Paulo Faculdade de Medicina Hospital das Clínicas São Paulo SP Brazil Departamento de Obstetrícia e Ginecologia, Hospital das Clínicas, Faculdade de Medicina, Universidade de São Paulo, São Paulo, SP, Brazil; Universidade Estadual de Campinas Faculdade de Ciências Médicas Departamento de Tocoginecologia Campinas SP Brazil Departamento de Tocoginecologia, Faculdade de Ciências Médicas, Universidade Estadual de Campinas, Campinas, SP, Brazil

## Key points

- Nonclassic congenital adrenal hyperplasia (NCAH) or late onset CAH is a genetic disease with late manifestations in women, usually in late childhood, adolescence or early adulthood.
- It is an important differential diagnosis of polycystic ovary syndrome.
- More than 90% of the time it is due to a deficiency of the 21-hydroxylase enzyme. Other less frequent causes are mutations in genes affecting the 11-beta-hydroxylase or 3-beta-hydroxysteroid dehydrogenase enzymes.
- Its diagnosis is particularly important in women who desire pregnancy.
- Treatment among women with hyperandrogenism without reproductive desire can be directed toward controlling clinical manifestations.

## Recommendations

- We recommend screening for NCAH due to 21-hydroxylase (CYP21A2) deficiency in adolescents and adult women who present signs and symptoms of hyperandrogenism (Level of Evidence A).
- The diagnosis of NCAH is excluded by measuring baseline 17-OHP < 2 ng/mL (<200 ng/dL). Values between 2 and 10 ng/mL (200 ng/dL and 1,000 ng/dL) indicate the need for a stimulus test with adrenocorticotropic hormone (ACTH) to confirm the diagnosis. When available, the test is performed by measuring 17-OHP at baseline and 60 minutes after intravenous or intramuscular administration of 250 μg of synthetic ACTH. If baseline or post-stimulus 17-OHP is > 10 ng/mL (1,000 ng/dL) and, more frequently > 15 ng/mL (1,500 ng/dL), the diagnosis of NCAH is established (Level of Evidence A).
- When possible, we recommend CYP21A2 genotyping in women presenting 17-OHP values > 10 ng/mL (1,000 ng/dL) with the aim to confirm the diagnosis and for pre-conception counseling (Level of Evidence B).
- We recommend the use of oral contraceptives (OC) associated or not with antiandrogens as the first option for treating androgenic manifestations and regularizing menstrual cycles in women who do not wish to become pregnant (Level of Evidence A).
- We recommend the use of glucocorticoids in cases where conventional treatment for hirsutism is ineffective or poorly tolerated or when the post-ACTH stimulus cortisol response is less than 18 mcg/dL (Level of Evidence C).
- We recommend the use of glucocorticoids that do not cross the placental barrier, such as hydrocortisone, prednisolone or prednisone for patients unable to become pregnant spontaneously or with ovulatory dysfunction and who are not at risk of affected fetuses (Level of Evidence B).
- In this situation, glucocorticoid treatment must be continued during pregnancy to avoid miscarriages (Level of Evidence C).
- We recommend combining ovulation inducers such as clomiphene citrate, letrozole (off-label) or gonadotropins if glucocorticoids are not sufficient to restore ovulation (Level of Evidence B).
- Genetic counseling for the couple is indicated to evaluate genotyping of the partner of women with the nonclassic form to detect a serious mutation (Level of Evidence C).
- We recommend referring pregnant women at risk of having female fetuses with genital defects to specialized tertiary and quaternary care centers (Level of Evidence D).

## Background

Congenital adrenal hyperplasia (CAH) comprises a group of autosomal recessive disorders associated with deficient biosynthesis of adrenal steroids whose clinical and laboratory expression depend on the enzyme involved. The most common enzymes are 21-hydroxylase (CYP21A2), 11β-hydroxylase (CYP11B1) and 17α-hydroxylase (CYP17A1).^(1,2)^ The first corresponds to more than 90% of cases, presenting 20%-50% of residual enzymatic activity in its nonclassic form (NCAH).^(1,3)^ This Position Statement will preferably address NCAH resulting from 21-hydroxylase deficiency, the most frequent and important differential diagnosis of polycystic ovary syndrome (PCOS).

## How prevalent is NCAH?

The prevalence of NCAH due to 21-hydroxylase deficiency in the general population is estimated at 1/1,000-2,000 in Anglo-Saxons, and 1/100 in Ashkenazi Jews and some populations from the Middle East and the Indian subcontinent. In women with hyperandrogenism, the prevalence ranges between 1% and 10%, depending on the ethnicity of the population studied (Level of Evidence C).^(4)^

## What are the manifestations of NCAH?

The main manifestations are premature pubarche (60%-90%), acne (30%), hirsutism (60%-80%), alopecia (2%-8%), clitoromegaly (6%-20%), menstrual irregularity (56%), primary amenorrhea (9%), chronic anovulation (30%-50%), infertility (10%-30%) and miscarriage (25%).^(4)^

## What is the deficiency in NCAH? Why are clinical manifestations variable?

Humans have two CYP21A genes: a non-functional pseudogene (CYP21P or CYP21A1) and an active gene (CYP21 or CYP21A2), both located on the short arm of chromosome 6. The mutation of this gene will condition the percentage of enzyme activity of the 21-hydroxylase, which, in turn, determines the severity of the disease. In nonclassic forms, 21-hydroxylase presents approximately 20%-50% of its activity. Thus, the phenotype is variable depending on the degree of enzymatic deficit, with a good correlation between the genotype and the phenotype documented in around 98% of cases. There are many polymorphisms in the CYP21A2 gene with normal enzymatic function. Most patients are compound heterozygotes, that is, they present different mutations in each of the alleles, and the clinical form is determined by the allele with the greatest enzymatic activity. Carriers have one normal allele and one mutated allele and do not have clinical disease, although they may present discrete biochemical changes.^(5)^

Specific mutations in the CYP21A2 gene are expressed by 21-hydroxylase deficiency and a defective conversion of 17α-OH-progesterone (17-OHP) into 11-deoxycortisol, generating a reduction in cortisol synthesis. Hypocortisolism leads to an increase in adrenocorticotropic hormone (ACTH) with consequent bilateral adrenal hyperplasia, accumulation of cortisol precursors (progesterone and 17α-OHP) and a shift towards the androgen production pathway (DHEA, androstenedione and testosterone).^(1,6,7)^

**Figure 1:**
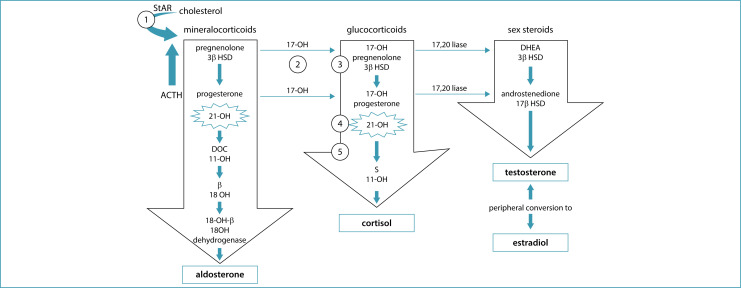
Adrenal steroidogenesis: the 5 steps necessary for cortisol production are shown in numbers. 1 = 20,22-desmolase; 2 = 17-hydroxylase (17-OH); 3 = 3β-hydroxysteroid dehydrogenase (3β HSD); 4 = 21-hydroxylase (21OHD); 5 = 11β-hydroxylase (11-OH). In the first step of adrenal steroidogenesis, cholesterol enters the mitochondria via the steroid hormone carrier protein (StAR). ACTH stimulates cholesterol cleavage, a rate-limiting step in the adrenal steroidogenesis

Source: Adapted from Yau et al. (2022).^(12)^

In 11β-hydroxylase deficiency, a less frequent form of CAH, but generally more severe, there is also excessive production of androgens, but with an increase in the immediate precursors of CYP11B1, 11-deoxycortisol (compound "S") and 11-deoxycorticosterone (DOC).^(8,9)^ As 17-OHP may also be elevated in these cases, it is important to confirm the etiological diagnosis. Deficiency of 17α-hydroxylase prevents the formation of glucocorticoids and sex hormones and promotes the accumulation of mineralocorticoid precursors (DOC) and corticosterone (Figure 1).^(10,11)^

## What is its pathophysiology?

In contrast to classic CAH, the nonclassic form is associated with partial activity of the 21-hydroxylase enzyme: there is no salt loss, anatomical changes are not evident at birth and biochemical changes are less pronounced. In general, the onset of clinical manifestations is peripubertal, with early pubarche, acne and accelerated bone age. In adolescent and adult women, the presence of hirsutism occurs in around 60%, oligomenorrhea in 54% and acne in 33%.^(3,4,13)^

Enzymatic deficiencies of 11β-hydroxylase and 17α-hydroxylase, rare in the NCAH, can occur together with arterial hypertension, although only 11β-hydroxylase deficiency is associated with hyperandrogenism, precocious pseudopuberty and advancing bone age. The deficiency of 17α-hydroxylase manifests itself in the post-pubertal period with primary amenorrhea, absence of secondary sexual characteristics and eunuchoid proportions (hypergonadotropic hypogonadism).^(10,11)^

Polycystic ovarian morphology is a frequent finding in women with NCAH; ovarian dysfunction may contribute to excess androgens in these women, and ovarian suppression improves clinical and laboratory hyperandrogenism^(14)^ (Level of Evidence B). Exaggerated secretion of progesterone and androgens could lead to dysfunction in the hypothalamic-pituitary-ovarian axis through an increase in the frequency of GnRH pulses with hypersecretion of luteinizing hormone (LH), contributing to excess ovarian androgens.^(4)^

## How to diagnose NCAH? Who should be investigated?

The investigation of NCAH is indicated in children with premature pubarche or accelerated growth rate and advanced bone age and in women with infrequent menstrual cycles associated with manifestations of hyperandrogenism. In these situations, dosing 17-hydroxyprogesterone (17-OHP) in the morning is indicated. The exam should preferably be performed in the follicular phase of the menstrual cycle in women who menstruate. The diagnosis is excluded by measuring baseline 17-OHP < 2 ng/mL (<200 ng/dL). Values between 2 and 10 ng/mL (200 ng/dL and 1,000 ng/dL) indicate the need for an ACTH stimulation test to confirm the diagnosis. The test is performed by measuring 17-OHP and cortisol at baseline and 60 minutes after intravenous or intramuscular administration of 250 μg of synthetic ACTH analogue (cortrosyn). If baseline or post-stimulus 17-OHP is > 10 ng/mL (>1,000 ng/dL and, more frequently > 15 ng/mL [>1,500 ng/dL]), the diagnosis of NCAH is established.^(4,15)^ (Level of Evidence A)

Given the difficulties in performing the cortrosyn test and low availability, a new cutoff value for 17-OHP was recently proposed to differentiate between PCOS and CAH, of 5.4 ng/mL, below which CAH would be ruled out.^(16)^

CYP21A2 genotyping is not considered necessary for routine diagnosis, but is recommended for patients with reproductive desire and circulating concentrations of 17-OHP above 10 ng/mL (30 nmol/L) with the aim to confirm the diagnosis and identify the presence of severe alleles of the disease that could lead to a higher risk of the classic form of the disease in the offspring of patients with NCAH^(4)^ (Level of Evidence B).

The main differential diagnosis of NCAH due to 21-hydroxylase deficiency is with PCOS. The prevalence of NCAH among women with hyperandrogenism is 1%-10%, much lower than the prevalence of PCOS, 50%-80%.^(17)^

## How should the treatment be carried out?

The aim of treatment is to regularize cycles and reverse the clinical manifestations of hyperandrogenism (acne, hirsutism, alopecia) and/or restore fertility. The use of combined oral contraceptives (COC) associated or not with antiandrogens is indicated for the treatment of androgenic manifestations and regularization of menstrual cycles. Antiandrogens are only recommended for patients not at risk of pregnancy, and the guarantee of an effective contraceptive method in those with an active sexual life because of the risk of inhibiting the sexual differentiation of male fetuses. Cyproterone acetate is a good option for treating NCAH due to 21-hydroxylase deficiency, used at a dose of 25-50 mg/day in the first 10 days of the cycle or contraceptive pack.^(18)^ Spironolactone is another antiandrogenic that can be used at an average dosage of 100 mg per day; its initial dose should be 25 mg, with a progressive increase to minimize side effects, reaching 200 mg/day. Although there is a hypothetical risk of salt loss due to the antimineralocorticoid effect of spironolactone in women with NCAH, this adverse effect has not been described.^(4,19)^ Compared to glucocorticoids, although not as effective in reducing androgen levels, cyproterone acetate is more effective in the treatment of hirsutism due to its peripheral action in blocking androgens^(20)^ (Level of Recommendation A).

In cases where conventional treatment for hirsutism is ineffective or poorly tolerated or when the post-ACTH stimulus cortisol response is less than 18 mcg/dL, the use of glucocorticoids may be indicated.^(19)^ In children with premature pubarche, treatment is generally carried out using hydrocortisone 10 to 15 mg/m^2^ divided into three daily doses, and may be replaced by the treatment of adult women sometime after menarche^(1,2)^ (Level of Evidence B). In adult women, hydrocortisone (15-25 mg/day, divided into 2-3 daily doses), prednisolone (4-6 mg, divided into 2 daily doses), prednisone (5-7.5 mg/day, divided into 2 doses daily) or dexamethasone (0.25-0.5 mg/day once a day) can be used with similar efficacy^(1,3)^ (Level of Evidence C).

Follow-up seeks to evaluate the improvement in androgenic manifestations and the reduction in serum androgen levels with treatment. However, circulating levels of 17-OHP may not normalize even with adequate doses of glucocorticoids and should not be considered as good control parameters during treatment.^(4)^

## What are the repercussions of NCAH on fertility?

Nonclassic congenital adrenal hyperplasia can manifest itself in women from puberty through signs of hyperandrogenism with regular and ovulatory cycles or even with infrequent cycles and infertility. Carriers of NCAH can also be asymptomatic.^(21)^ In 13% of cases, infertility is the symptom that leads to investigation and diagnosis.^(22)^ As opposed to the classic form of the disease, in this milder form, fertility rate appears to be slightly decreased. In a longitudinal study of 95 women with NCAH, 57.2% of them became pregnant without any specific hormonal treatment.^(23)^

The mechanism by which increased androgen production in NCAH interferes with ovulation is not yet well understood. One theory focuses on the direct effect of hyperandrogenemia on the GnRH pulse generator. Excess androgens affect hypothalamic sensitivity to progesterone, resulting in an increase in the frequency of GnRH pulses, which favors LH hypersecretion. In turn, this hypersecretion can initiate and maintain a vicious circle in which LH stimulates ovarian theca cells, further exacerbating the consequences of androgen secretion of adrenal origin. The normalization of LH levels and their response to GnRH pulses with corticosteroid treatment corroborates this mechanism.^(21)^ Furthermore, previous studies demonstrate that the risk of miscarriage decreased by more than 70% after diagnosis of the disease and initiation of treatment with glucocorticoids, going from 26% to 6%^(23,24)^ (Level of Evidence C).

In women with NCAH who are unable to become pregnant spontaneously or have ovulatory dysfunction, treatment with glucocorticoids that do not cross the placental barrier, such as hydrocortisone, prednisolone or prednisone, is recommended^(1)^ (Level of Evidence B). Recommended doses of hydrocortisone range from 20-25 mg/day and 2.5-5 mg/day of prednisone, although there are no prospective studies evaluating the benefits in relation to reducing early pregnancy loss and improving gestational outcomes.^(4)^ Dexamethasone should not be used in these situations, as it crosses the fetoplacental barrier, potentially causing deleterious effects on the intellectual development of the fetus in the long term^(25,26)^ (Level of Evidence D). Treatment with glucocorticoids must be maintained during pregnancy to avoid miscarriages^(1,4,23,27)^ (Level of Evidence A). The clinical criterion for evaluating therapeutic efficacy before pregnancy is the regularization of menstrual cycles, while biological criteria are the normalization of testosterone and androstenedione.^(28)^

If treatment with glucocorticoids is not sufficient to normalize gonadotrophic function, ovulation inducers such as clomiphene citrate or letrozole can be associated.^(23)^ For more refractory cases, injectable gonadotropins can be used carefully to avoid a multifollicular response and the risk of twin pregnancy^(21)^ (Level of Evidence B).

## What should prenatal diagnosis and treatment of 21-hydroxylase deficiency be like?

In women diagnosed with NCAH, almost 70% are compound heterozygotes, carrying one allele that causes the nonclassic form and one for the classic form of the disease. The mildest mutation leads to the manifestation of the nonclassic form. However, the offspring of these patients have a 50% chance of inheriting the allele that leads to the classic form of the disease. Theoretically, without genotyping, if one of the parents has NCAH, there is a risk of around 1:250 of having a child with the classic form of the disease,^(15,29)^ although retrospective studies show a prevalence around 1.5%-2.5%.^(23,24)^ Therefore, genetic counseling for the couple is indicated to evaluate the genotyping of the partner of women with the nonclassic form to detect a serious mutation^(23)^ (Level of Evidence C). As the formation of genitalia occurs around the ninth week of pregnancy, the collection of fetal DNA in a maternal peripheral blood sample can be performed around the sixth week of pregnancy to identify the presence of the Y chromosome and exclude male fetuses from prenatal dexamethasone treatment.^(30)^

In cases of risk of birth of female fetuses with genital defects, the prenatal treatment protocol with glucocorticoids is considered experimental and only authorized in tertiary and quaternary care centers that have defined protocols approved by ethics committees. The aim of prenatal treatment is to prevent genital virilization of the fetus. In this situation, some authors recommend the use of dexamethasone due to its ability to cross the placenta and because it is not deactivated by placental 11β-OH steroid dehydrogenase and only minimally binds to cortisol-binding globulin in the mother's blood. Dexamethasone decreases elevated fetal androgen production by suppressing fetal ACTH secretion. Treatment may be discontinued if the karyotype reveals a male fetus or DNA analysis reveals an unaffected female fetus.^(31)^ There are few studies evaluating the long-term outcomes of prenatal use of dexamethasone to prevent virilization of female fetuses. A 2020 systematic review and meta-analysis included 18 cohort studies evaluating the use of dexamethasone in pregnancies at 6-10 weeks. The meta-analysis of these studies showed a reduction in the virilization of female fetuses treated with dexamethasone in early pregnancy. There was no significant difference in relation to physical outcomes of newborns, such as weight and height, as well as cognitive functions and behavioral or temperament changes. However, these results must be evaluated with caution given the observational nature of the studies with small samples and moderate quality in over 50% of them.^(30)^ In relation to maternal safety, there may be a risk of weight gain, appearance of stretch marks, edema and sleep and mood disorders. On the other hand, no increased risk of hypertension, gestational diabetes or fetal loss was reported.^(31)^ Note that there are studies reporting orofacial defects in children exposed to glucocorticoids during pregnancy.^(32,33)^ Treatment with dexamethasone also has been suggested in cases of women who have already had an affected child and are pregnant with the same partner. In reference centers, dexamethasone would be initiated to suppress fetal androgen production before the onset of urogenital organogenesis at the ninth week of pregnancy^(1,3,30)^ (Level of Evidence D). Another alternative would be to perform in vitro fertilization with preimplantation genetic testing for monogenic diseases and the transfer of only embryos unaffected by the disease.^(30)^

## Final considerations

This text was prepared by the National Specialized Commission on Endocrine Gynecology of Febrasgo. Its aim is to update and assist gynecologists, obstetricians and primary care physicians in the diagnosis and treatment of NCAH in women.

## National Specialized Commission on Endocrine Gynecology of the Brazilian Federation of Gynecology and Obstetrics Associations (FEBRASGO)

**President:**

Cristina Laguna Benetti Pinto

**Vice president:**

Ana Carolina Japur de Sa Rosa e Silva

**Secretary:**

José Maria Soares Júnior

**Members:**

Andrea Prestes Nácul
Daniela Angerame Yela
Fernando Marcos dos Reis
Gabriela Pravatta Rezende
Gustavo Arantes Rosa Maciel
Gustavo Mafaldo Soares
Laura Olinda Rezende Bregieiro Costa
Lia Cruz Vaz da Costa Damásio
Maria Candida Pinheiro Baracat Rezende
Sebastião Freitas de Medeiros
Tecia Maria de Oliveira Maranhão
Vinicius Medina Lopes
